# Detection of Rice Pests Based on Self-Attention Mechanism and Multi-Scale Feature Fusion

**DOI:** 10.3390/insects14030280

**Published:** 2023-03-13

**Authors:** Yuqi Hu, Xiaoling Deng, Yubin Lan, Xin Chen, Yongbing Long, Cunjia Liu

**Affiliations:** 1 College of Electronic Engineering (College of Artificial Intelligence), South China Agricultural University, Guangzhou 510642, China; 2 National Center for International Collaboration Research on Precision Agricultural Aviation Pesticide Spraying Technology, Guangzhou 510642, China; 3 Guangdong Laboratory for Lingnan Modern Agriculture, Guangzhou 510642, China; 4 Guangdong Engineering Technology Research Center of Smart Agriculture, Guangzhou 510642, China; 5 Unmanned Vehicles Department of Aeronautical and Automotive Engineering, Loughborough University, Leicestershire LE11 3TU, UK

**Keywords:** rice pest detection, YOLOv5, Swin Transformer, BiFPN, self-attention

## Abstract

**Simple Summary:**

Various types of rice pests cause huge losses to rice production every year in China. In this paper, a deep neural network for pest detection and classification via digital images is proposed. The targeted optimization is improved for the pest characteristics. Our experiments determined that our model has a higher accuracy and detection speed compared with other methods. In addition, it can be more widely used in pest detection surveys for various crops.

**Abstract:**

In recent years, the occurrence of rice pests has been increasing, which has greatly affected the yield of rice in many parts of the world. The prevention and cure of rice pests is urgent. Aiming at the problems of the small appearance difference and large size change of various pests, a deep neural network named YOLO-GBS is proposed in this paper for detecting and classifying pests from digital images. Based on YOLOv5s, one more detection head is added to expand the detection scale range, the global context (GC) attention mechanism is integrated to find targets in complex backgrounds, PANet is replaced by BiFPN network to improve the feature fusion effect, and Swin Transformer is introduced to take full advantage of the self-attention mechanism of global contextual information. Results from experiments on our insect dataset containing Crambidae, Noctuidae, Ephydridae, and Delphacidae showed that the average mAP of the proposed model is up to 79.8%, which is 5.4% higher than that of YOLOv5s, and the detection effect of various complex scenes is significantly improved. In addition, the paper analyzes and discusses the generalization ability of YOLO-GBS model on a larger-scale pest data set. This research provides a more accurate and efficient intelligent detection method for rice pests and others crop pests.

## 1. Introduction

Rice is the world’s main food crop, feeding half of the world’s population and about two-thirds of China’s population [1]. According to the International Rice Research Institute, farmers lose an average of 37% of their rice production each year due to insect pests and diseases. Pests cause hundreds of millions of dollars in losses worldwide every year. Timely and accurate identification of pests can carry out targeted prevention and control work to reduce economic losses due to serious pests.

The traditional pest detection method is mainly manual identification. Agricultural technicians observe and identify pests with the naked eye using hand lenses and microscopes. This task requires continuous monitoring of crops. For large farms, this is a subjective, labor-intensive, and expensive task [2]. With the development of technology, traditional image processing techniques were used in the field of pest detection, and most of these methods manually design image features to identify specific species of pests [3,4,5,6]. Compared with manual visual detection methods, traditional image processing techniques can greatly improve the recognition efficiency and solve the problems of lack of agricultural experts and poor objectivity. However, due to the limitation of manual feature extraction, such algorithms only focus on the detection of specific pests in specific scenarios, lack generality, and have difficulty to meeting work needs in practical scenarios.

With the continuous development of computer vision and deep learning technology, more and more researchers are committed to combining artificial intelligence with the field of agriculture [7]. The pest detection algorithm based on deep learning does not need to design complex features artificially. It can automatically extract the features in the original image for identification, positioning, and counting. Classic algorithms include Faster RCNN [8], SSD [9], YOLOv3 [10], Cascade R-CNN [11]. Shen et al. [12] achieved the detection of food storage insects by combining an insect trapping device with Faster RCNN. Dai et al. [13] used an improved cascade R-CNN with high-definition cameras in the wild to achieve citrus psyllid detection. Rong et al. [14] used Mask R-CNN to identify and count field pests of yellow plate, and effectively solved the problem of inaccurate small target recognition by improving the FPN structure in the feature extraction network. Various pest detection methods based on deep learning have the characteristics of fast detection speed, high detection accuracy, and strong generality, which provide a new method for crop pest detection.

At present, there are few effective machine learning detection algorithms for rice pests, due to the problems such as high similarity between classes, large changes in target scale, and complex backgrounds that are difficult to detect. Sethy et al. [15] proposed a simple method to quantify the degree of infection of Rice Brown Plant Hopper (RBPH, *Nilaparvata lugens Stal*, Hemiptera: Delphacidae). Median filtering and K-mean clustering were used to segment the RBPH area in the image, and then the ratio of infested RBPH area and total area was compared to quantify the degree of infection. Qing et al. [16] collected images through a self-made handheld image acquisition device, and detected and counted white-backed planthoppers through a three-layer detector including AdaBoost classifier, SVM classifier, and threshold judgment of multiple features, with a detection rate of 85.2%. However, this method needs to be manually set the threshold, and the model lacks universality as the detection target is only for rice planthoppers. Liu et al. [17] used a global contrast region-based method to calculate a saliency map for locating pest objects. Then, based on the saliency map for extracting the boundary containing the target, Liu et al. sent the target area an image adjusted to a fixed size for the DCNN network for classification, identified 12 types of typical rice pests, and finally achieved a mean accuracy precision (mAP) of 0.95. However, the basis of the feasibility of this method is the sharp contrast between the pest and the background, and it cannot handle the situation that the target is not salient enough or has occlusion. In addition, the optimal threshold based on saliency map segmentation needs to be determined by experiments. An inappropriate threshold will also affect its accuracy. He et al. [18] used a dual-layer target detection algorithm to detect brown planthoppers. Both layers of networks are fast RCNN, but each layer selects different feature extraction networks. Experiments show that the detection result of a dual-layer algorithm is obviously better than that of single-layer detection algorithm. This method provides a new way for pest detection, but the model structure of fast RCNN is very large, and the detection speed of the algorithm is slow.

Although many researchers have performed a lot of work in the detection of rice pests, there is still a lack of intelligent and effective rice pest detection algorithms under open fields based on deep learning. The existing algorithms still need to be further improved in terms of the types of pests that can be detected and the requirements for the detection scene. In addition, agricultural applications are often used outdoors, with limited available resources, and lower computing power often results in lower detection speed. Therefore, it is necessary to explore an accurate and efficient rice pest identification algorithm. This study improves the YOLOv5 network model by integrating the GC attention mechanism, enhancing the ability of the network to extract image features, adding a detection head to increase the detection scale range, and introducing BiFPN and Swin Transformer to improve the detection performance. Based on the above improved methods, the YOLO-GBS algorithm is proposed for the detection of rice pests.

The YOLO-GBS has great potential for practical applications in the field of pest screening, surveyors, and management. For pest screeners, the model can provide accurate and efficient identification of pests, greatly reducing the workload and improving the accuracy of pest identification. For surveyors, the model can help identify pests in the early stages of infestation and monitor their spread, thus helping to prevent and control pest outbreaks. For managers, the model can provide valuable information for decision-making, such as identifying areas that require targeted pest control measures and tracking the effectiveness of control efforts. Overall, the YOLO-GBS model can greatly enhance the efficiency and effectiveness of pest management practices, which is of great importance for agriculture.

## 2. Materials and Methods

### 2.1. Image Dataset

**Figure 1:**
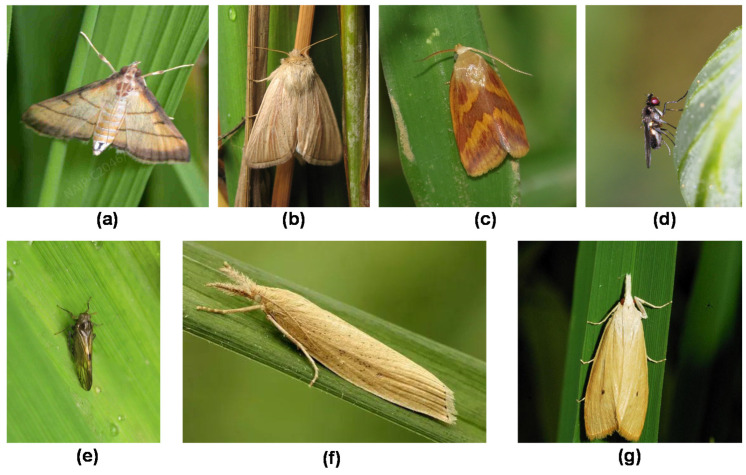
Samples of seven types of rice pests: (**a**) Rice leaf roller, (**b**) pink rice borer, (**c**) rice leaf caterpillar, (**d**) paddy stem maggot, (**e**) plant hopper, (**f**) Asiatic rice borer, (**g**) yellow rice borer.

**Figure 2:**
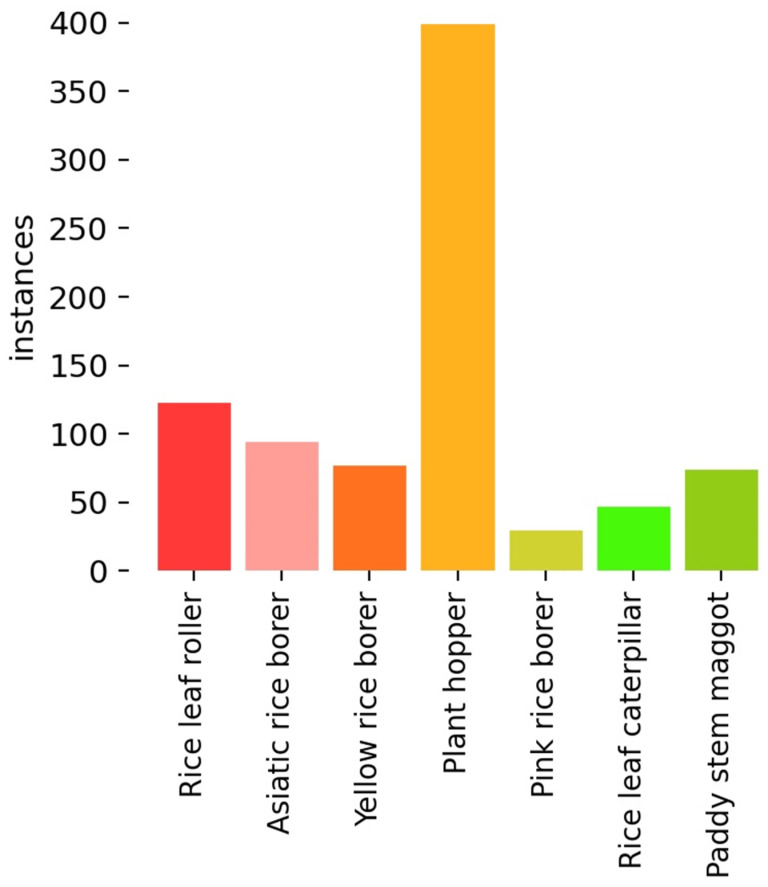
The number of labels of each category.

The dataset in this research comes from the IP102 dataset [19] and web crawler. IP102 is a large-scale dataset for pest identification, which contains more than 75,000 images with 102 categories. It was proposed in 2019 and so far still has the largest pest data set. In this work, seven types of adult pests of rice were selected, including rice leaf roller (*Cnaphalocrocis medinalis*, Lepidoptera: Crambidae), pink rice borer (*Sesamia inferens*, Lepidoptera: Noctuidae), rice leaf caterpillar (*Naranga aenescens Moore*, Lepidoptera: Noctuidae), paddy stem maggot (*Hydrellia griseola*, Diptera: Ephydridae), plant hopper (*Nilaparvata lugens Stal*, *Sogatella furcifera Horvath*, *Laodelphax striatellus Fallén*, Homoptera: Delphacidae), Asiatic rice borer (*Chilo suppressalis Walker*, Lepidoptera: Crambidae), and yellow rice borer (*Scirpophaga incertulas Walker*, Lepidoptera: Crambidae). The samples of the seven categories are shown in Figure 1. Due to the problems such as the duplication of images and low resolution in the IP102 dataset, the images were manually screened and cleaned, leaving 684 images. Due to the prominent long-tail phenomenon of the dataset, 181 pictures were collected by web crawlers to balance the data, and finally, a total of 865 images of pests were obtained. LabelImg software v1.8.1 was used for manual annotation to obtain a ground truth for subsequent training. The original dataset was divided into the training set, validation set, and test set with the ratio of 6:2:2. Data enhancement adopted online enhancement. Before each epoch training, each image was enhanced according to the set probability. The enhancement strategies included mosaic, clipping, horizontal flipping, translate, hue, saturation, and brightness adjustment. The number of labels for each category is shown in Figure 2.

### 2.2. The Proposed Method (YOLO-GBS)

**Figure 3:**
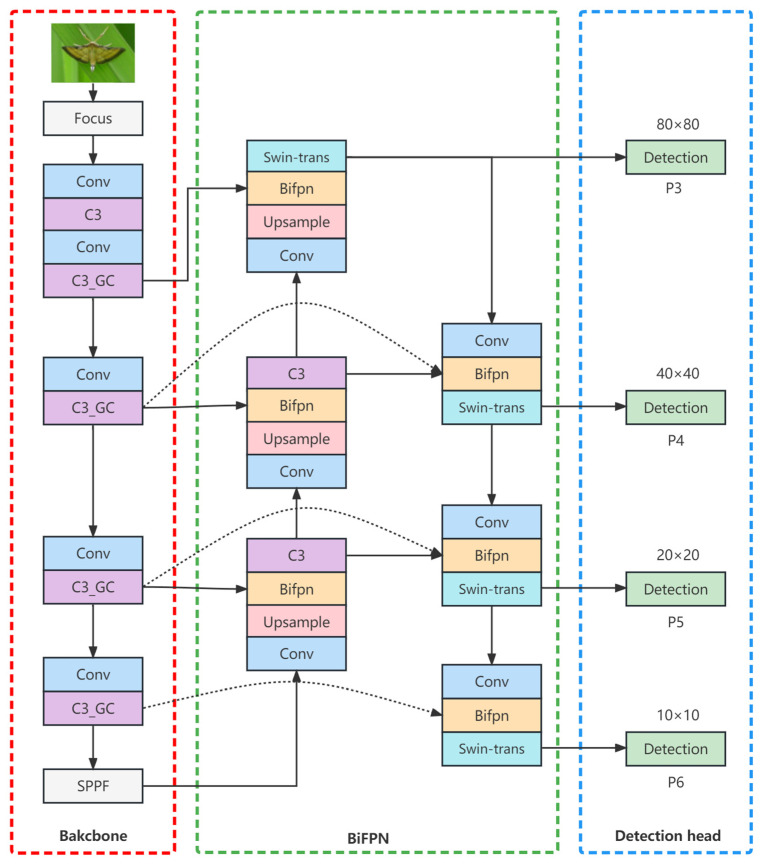
Structure diagram of YOLO-GBS.

The YOLO [20] series of network models are widely used in various fields due to their excellent performance in speed and accuracy. YOLOv5 was released and open sourced by ultralytics in 2020. As the best single-stage target detection model so far, the backbone of the network is composed of the classic CSPDarknet53 structure, Focus module, and SPP module, using PANet as the neck network, as well as head, using the classic YOLO detection head. By controlling the depth and width of each module in the network, YOLOv5 can be divided into four different models, including YOLOv5s, YOLOv5m, YOLOv5l, and YOLOv5x, and the model scales increase sequentially. Considering the miniaturization and real-time requirements for agriculture applications, this study selected YOLOv5s model with the smallest number of parameters and the fastest inference speed as the baseline model. After applying the YOLOv5s model to rice pest detection, it was found that due to the huge difference between pest detection and general target detection, YOLOv5s model was not found satisfactory in the detection of complex scenes, dense small targets, and occluded targets. In view of this situation, an improved algorithm called YOLO-GBS (YOLO with GCNet, BiFPN and Swin Transformer) was proposed in the study to adapt to specific rice pest detection tasks, and the structure of YOLO-GBS is shown in Figure 3.

Considering the characteristics of slight differences in appearance, large changes in the size of pest images, and the low accuracy of the existing recognition algorithm, this study introduced the GC attention mechanism to improve the feature extraction effect, used BiFPN to replace the original PANet feature fusion network to increase the richness of network features after fusion, adopted Swin Transformer to replace part of the convolutional structure in the deep network to improve the ability to extract global information, and increased the number of detection heads to increase the detectable target scale range.

#### 2.2.1. Global Context Attention Mechanism

For agricultural images, complex backgrounds and non-salient targets often bring difficulties to the recognition of the model. To better extract target features and reduce the interference caused by non-target areas, the global context attention mechanism (GC) [21] was introduced to the main feature extraction module C3, which is a self-attention-based attention mechanism that combines the capture ability of non-local network [22] for long-range dependencies and SENet [23] lightweight. The GC attention mechanism consists of three steps: (1) context modeling—using 1 × 1 convolution $W_{k}$ and softmax function to obtain attention weights through global attention pooling, and then obtaining global context features through pooling; (2) transformation—performing feature transformation through 1 × 1 convolution $W_{v}$, referring to the bottleneck design in SE block, controlling the parameter through a dimensionality-reduction layer with reduction ratio r, and using layer normalization to reduce the optimization difficulty; (3) feature fusion—fusing the global context features to each location.

The mathematical expression of GC attention is as follows:

(1) $$\begin{array}{c} z_{i}=x_{i}+W_{v2}ReLU\left(LN(W_{v1}\sum_{j=1}^{N_{p}}\frac{e^{W_{k}x_{j}}}{\sum_{m=1}^{N_{p}}e^{W_{k}x_{m}}}x_{j})\right) \end{array}$$

where $N_{p}$ is the number of positions in the feature map, for pictures $N_{p}$ = height × width. $\alpha_{j}$ = $\frac{e^{W_{k}x_{j}}}{\sum e^{W_{k}x_{m}}}$ is the weight for global attention pooling, and δ(·) = $W_{v2}ReLU(LN\left(W_{v1}(\cdot )\right))$ denotes the bottleneck transform. $x_{i}$ represents input features, and $z_{i}$ represents GC block output features.

**Figure 4:**
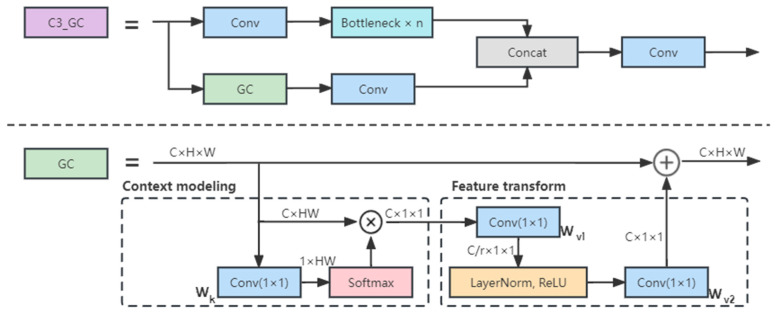
Structure diagram of C3_GC and GC block.

The structure of the C3_GC and GC block is shown in Figure 4.

#### 2.2.2. Multi-Scale Feature Fusion

For the target detection task, the fusion of multi-scale features can greatly reduce the loss of features in the convolution process and improve the detection effect. At present, the main feature fusion networks are FPN [24], PANet [25], NAS-FPN [26], BiFPN [27], etc. BiFPN simplifies the network while retaining the bottom-up and top-down bidirectional integration of PANet, and deletes the nodes with only one input side because these nodes make little contribution to the network. A skip connection was added between the original input node and the output node to achieve the purpose of fusing more, richer features. In feature fusion, learnable weights are introduced to learn the importance of different input features, and thus adjust the contribution of each input feature.

**Figure 5:**
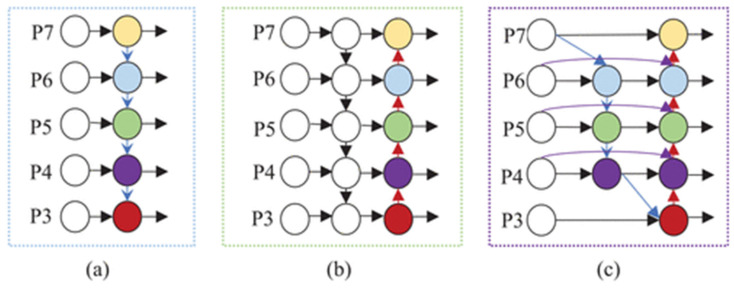
Feature fusion structural model diagrams: (**a**) FPN, (**b**) PAN, (**c**) BiFPN. P3-P7 represent features from different layers of the model.

Because of the above advantages, BiFPN was adopted to replace the original PANet of YOLOv5 in the neck network. The structural model diagrams of FPN, PAN, and BiFPN are shown in Figure 5. The FPN is one-way fusion, PANet adds bottom-up two-way fusion, and BiFPN adds skip structure and weight to each fusion feature.

#### 2.2.3. Swin Transformer

Transformer was originally used in the field of natural language processing and first proposed by Google in Attention as All You Need [28]. In 2020, Vision Transformer [29] brought the transformer to the computer vision field for the first time, and began to shine in this field. A large number of excellent networks such as DeiT [30], Swin Transformer [31], DETR [32], SETR [33], and GANsformer [34] came out one after another.

**Figure 6:**
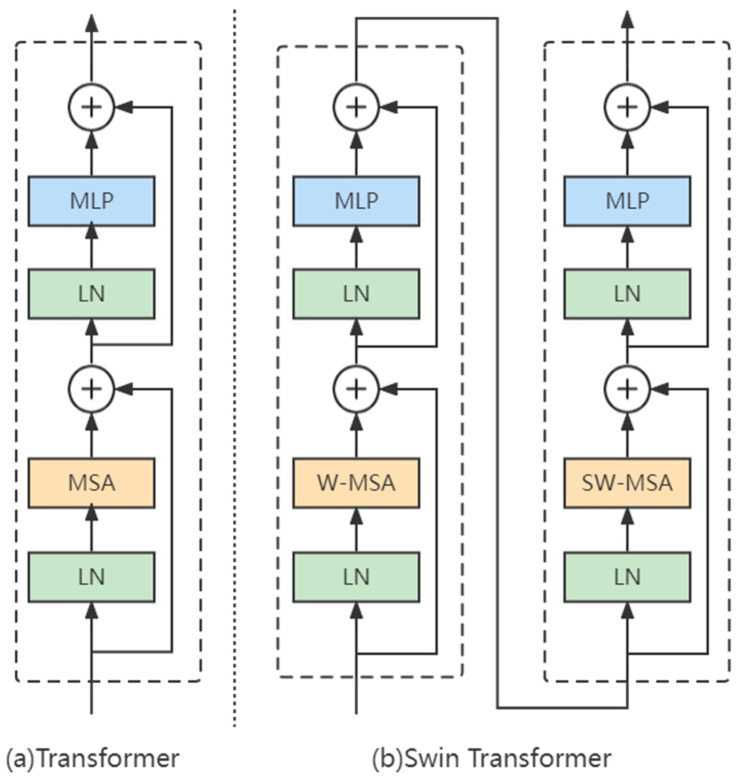
Structure diagram of (**a**) Transformer and (**b**) Swin Transformer. LN stands for Layer Normalization, MSA stands for Multi-head Self-Attention Mechanism, and MLP stands for Multi-Layer Perceptron.

In the standard Transformer structure, the global self-attention is calculated every time, which is very computationally expensive for high-resolution images. In order to improve efficiency, a window-based Multi-head Self-Attention (W-MSA), which divides the original image into multiple non-overlapping windows and performs self-attention operation inside each small window, is introduced in Swin Transformer, and a Shifted Windows Multi-Head Self-Attention (SW-MSA) is also introduced to Swin Transformer to make up for the information transfer between different windows. By moving the position of the window on the previous layer, a connection is introduced between the non-overlapping windows on the previous layer, thereby greatly increasing the receptive field. The comparison between Transformer and Swin Transformer is shown in Figure 6.

A convolutional neural network (CNN) is good at extracting shallow features, but not good at capturing global information and context in deep features, which are exactly what transformers are good at. In this paper, considering that there are many small targets and occluded targets (part of the body is obscured) in rice pest detection application, such features are easily ignored in the convolution process; the 3 × 3 convolution of the C3 module in the YOLOv5s neck is replaced by a Swin Transformer block, expecting to achieve better classification and localization effects through better contextual information extraction.

#### 2.2.4. Additional Detection Head

**Figure 7:**
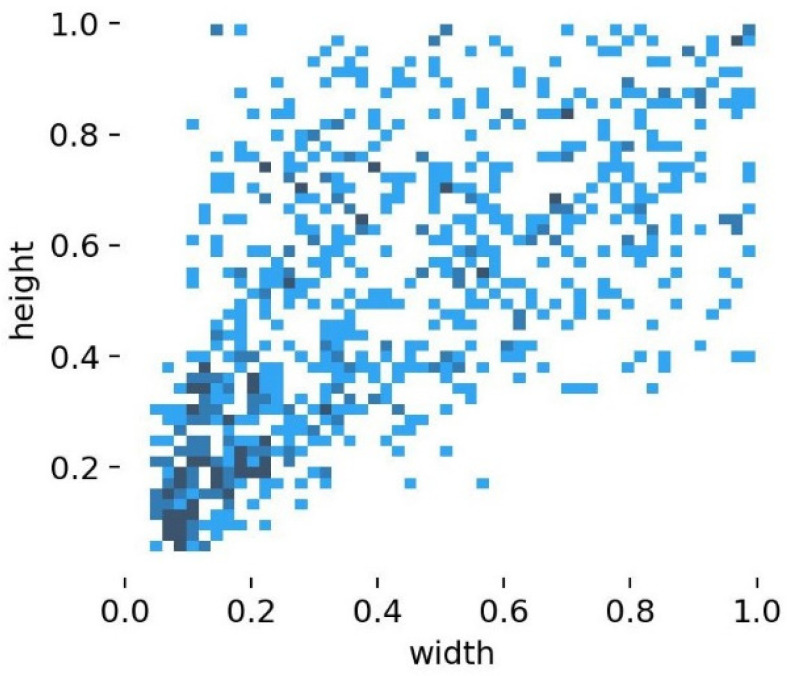
Target width and height distribution map.

The scale of agricultural pest targets varies widely. Figure 7 shows a visualization of the width and height of the pest targets in the dataset as a proportion of the total image. The horizontal axis represents the ratio of the target’s width to the total width of the image, while the vertical axis represents the ratio of the target’s height to the total height of the image. It can be found in Figure 7 that the target scales are widely distributed in various ranges. In this study, to better capture various targets ranging from large (such as various moths) to small (such as the rice planthopper), an additional detector head P6 is added, generated from a high-level and low-resolution feature map, with an output feature size of 10 × 10. This detection head is more sensitive to large-sized targets.

### 2.3. Experiment Environment and Model Evaluation

The hardware configuration for the experiments includes Intel(R) Core(TM) i7-10700 CPU @ 2.90 GHz and memory with 32 GB, NVIDIA RTX 3090 graphics card with 24 GB graphics memory. The software environment is Windows 10 Professional 64-bit operating system, CUDA version 11.1, CUDNN version 8.0.5, Python version 3.7 and PyTorch version 1.10.1.

In the experiment, the input image pixels were 640 × 640. The model was trained on self-built rice pest dataset for 300 epochs. The batch size was set to 32, and online data enhancement methods such as mosaic, mirroring, flipping, and brightness adjustment were used in the training process to enrich the background of the detected objects further and strengthen the cognition of the network model on pest characteristics. AdamW was used as the optimizer, the initial learning rate was set as $1\times 10^{-3}$, and the one-cycle linear learning rate was updated and optimized during the training process.

To measure the accuracy of the proposed method, the evaluation indicators were adopted such as Precision, Recall, Average Precision (AP), and mean Average Precision (mAP) as evaluation indicators; the formula of those indicators are shown as Equations (2)–(5):

(2) $$\begin{array}{c} Precision=\frac{TP}{TP+FP} \end{array}$$

(3) $$\begin{array}{c} Recall=\frac{TP}{TP+FN} \end{array}$$

(4) $$\begin{array}{c} AP=\int_{0}^{1}P(R)dR \end{array}$$

(5) $$\begin{array}{c} mAP=\frac{\sum AP}{N_{class}} \end{array}$$

where *TP* represents the number of true positive samples, *FP* represents the number of false positive samples, and *FN* represents the number of false negative samples. By drawing the curve showing that *P* changes with *R* in the interval 0–1, the value of *AP* can be calculated with the area under the curve, and the final *mAP* value can be obtained by averaging the *AP* values of each category.

## 3. Results

### 3.1. Ablation Studies

To verify the contribution of each proposed module to the overall performance of the network, ablation experiments were carried out on the dataset. The results of all ablation experiments are shown in Table 1, where it can be found that the performance improvement gradually increased from the YOLOv5s (baseline) to the final YOLO-GBS.

From Table 1, the model can capture a wider range of target scales by adding an additional detection head (YOLOv5s + P6). Although the number of model layers and calculation amount increased from 270 layers and 16.5 GFLOPs to 355 layers and 16.9 GFLOPs separately, the model recall was also greatly improved, which was 2.3% higher than that of YOLOv5s network, and the mAP also increased by 0.7%. The BiFPN feature fusion network (YOLOv5s + P6 + BiFPN) and the GC attention mechanism (YOLOv5s + P6 + BiFPN + GC) further improve the model’s ability to detect targets through better fusion and extraction of features, so that the model mAP increased by 0.3% and 1.1% respectively. YOLOv5s + P6 + BiFPN + GC + Swin Transformer, namely YOLO-GBS, which is the final solution network for this paper, achieved the best performance with it excellent global information and context acquisition ability. The mAP of the YOLO-GBS proposed in this study was 79.8% on the pest dataset, which was 5.4 percentage points higher than the original YOLOv5s on the same test set, indicating the feasibility and effectiveness of the improved model in this study. The proposed YOLO-GBS played a great role in detecting small targets, dense targets, and occluded targets.

**Figure 8:**
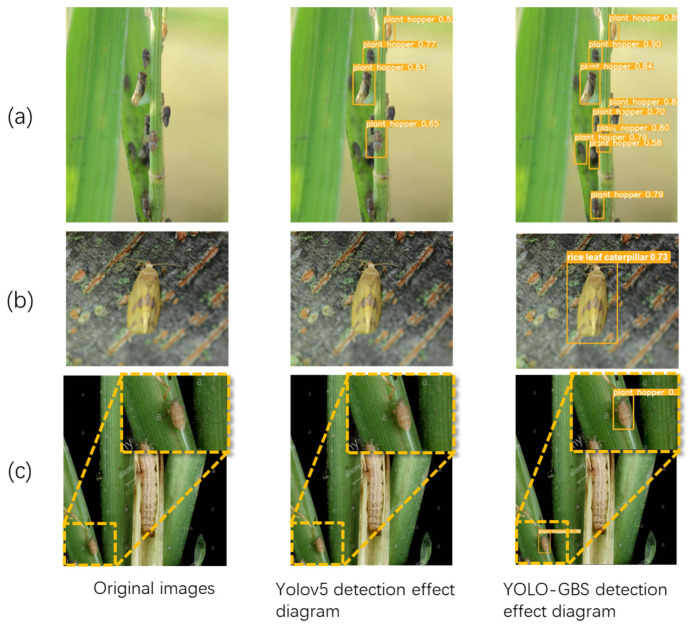
Comparison of detection results. (**a**) Occluded target, dense target. (**b**) Camouflaged target. (**c**) Small target. The image inside the dashed line is a zoom-in of the lower left corner of the original image.

To better show the effect of the improved model, YOLOv5 and YOLO-GBS models were compared with three typical complex scenarios in the test set, including dense and occluded target, small target, and camouflaged target, as shown in Figure 8. The left side shows the original image, the middle side shows the detection result of YOLOv5s, and the right side shows the detection results of the YOLO-GBS model. Obviously, YOLOv5s detection misses some pest targets, while the YOLO-GBS model recognizes all targets in typical complex scenes.

### 3.2. Comparison of Various Mainstream Networks

**Figure 9:**
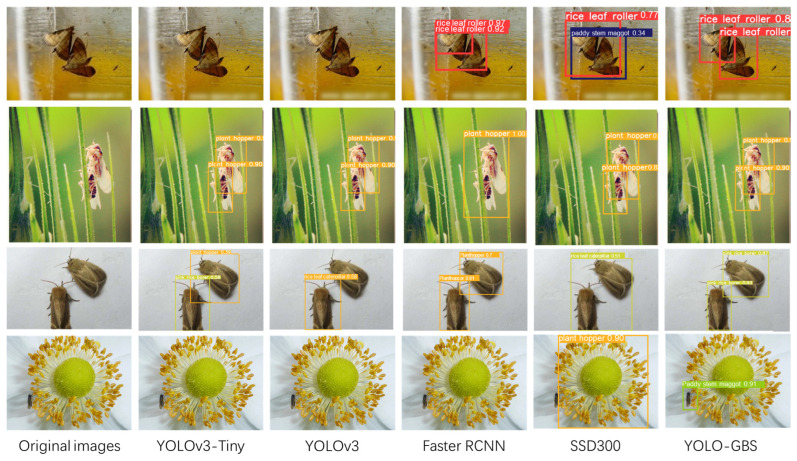
Visualization results.

To better verify the performance of the improved model, SSD300, YOLOv3, YOLOv3-tiny, and faster RCNN, which are the mainstream networks, were used to compare with YOLO-GBS, and the comparison performance is shown in Table 2. It can be seen that single image detection time of YOLO-GBS is 3.2 ms, second only to 1.9ms of YOLOv3-tiny, but the mAP is significantly ahead by 10%. The average accuracy of the improved YOLO-GBS is the highest among all the comparison models, 4.4 percentage points higher than the second place Faster RCNN, and the single image detection time is about 6 times faster. Considering both accuracy and speed, YOLO-GBS has the best comprehensive performance and can complete well in the task of detecting rice pests. The comparison visualization results are shown in Figure 9. In addition to YOLO-GBS, other models have some problems such as missed detection or bounding box positioning errors. Regarding the small target in the last row, only YOLO-GBS successfully detects and locates it accurately.

### 3.3. Model Generalization Capability

In order to further explore the generalization ability of the model in the detection of crop diseases and pests, the generalization ability of the model was evaluated using the unwashed IP102 dataset. Different from the previous training process, the dataset used this time includes 18,976 pest pictures of 102 classes and their corresponding annotation files. The dataset was divided into training set, validation set, and test set in the ratio of 6:2:2. The same YOLO-GBS network was trained again on this complex and diverse dataset, and the comparison testing results of different models are shown in Table 3.

It can be seen from Table 3 that YOLO-GBS can still effectively identify and classify pests even for larger and more complex data sets. Compared with other models, the proposed YOLO-GBS has the best mAP, indicating that the model has good generalization performance and may be further applied to various pests of various crops.

**Figure 10:**
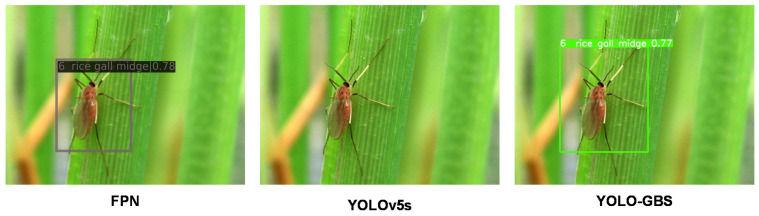
YOLOv5s did not detect the insect in the figure.

**Figure 11:**
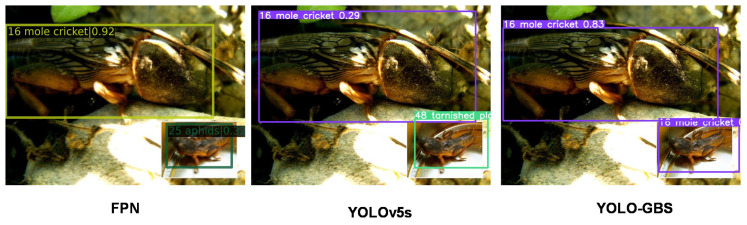
YOLOv5s and FPN misclassified the insect in the lower right corner of the figure; the correct classification is “16 mole cricket”.

**Figure 12:**
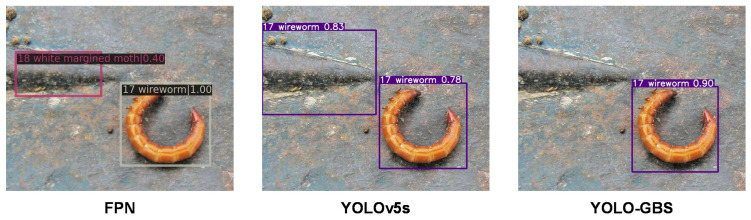
YOLO5s and FPN misdetection of the upper left background of the figure as insects.

The FPN and YOLO algorithms, which have higher accuracy rates among the above methods, are chosen to compare their effects with the methods in this paper. It can be seen that every insect in Figure 10, Figure 11 and Figure 12 can be detected by our method, and each presents good performance in terms of detection and classification accuracy.

### 3.4. Grad-CAM Visualisation

**Figure 13:**
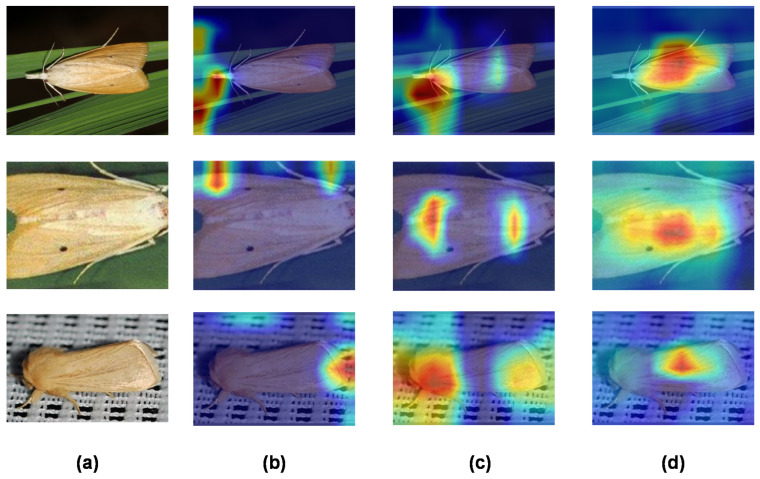
Visualization of the feature maps. (**a**) The original images; (**b**) the heatmaps of layer 10 in backbone; (**c**) the heatmaps of the BiFPN structure; (**d**) the heatmaps of the output layer.

This experiment used Gradient-weighted Class Activation Mapping (Grad-CAM) [35] to visualize the reasoning process, trying to both further explain the process of generating the results and analyzing and discussing the advantages and disadvantages of the model as well as future improvements. In order to show how the improved model makes decisions, each result at different layers was visualized using a heatmap drawn by Grad-CAM to show the regions of interest at different layers. Three examples were used to visualize the decision-making process, and some key Grad-CAM diagrams of the network were selected, including the tenth layer of the backbone network, the BiFPN structure, and the output layer. Although there is a certain degree of deviation between the hot spot of layer 10 shown in Figure 13b and the actual target shown in Figure 13a, after the weighted fusion of BiFPN structure and other layer features, the hot spot has been biased towards the actual target shown in Figure 13c. After the Swin Transformer structure, the final output layer was further optimized, as shown in Figure 13d, where the hot spot position displayed by the heat map is basically consistent with the real target.

## 4. Discussion

Although YOLO-GBS has achieved promising results, there are several issues that deserve attention. Firstly, due to the low image resolution of the public datasets used, there are often cases where the localization is accurate, but the classification is incorrect, especially in scenes where two types of worms have similar colors and can only be distinguished by their textures. Therefore, high-granularity insect classification remains a future research direction worth pursuing.

Secondly, to further realize unmanned pest detection and field application without network, the model may need to be deployed on various edge devices, such as high-definition cameras, various insect trapping devices, unmanned vehicles, etc. How to further reduce the demand for computing power will become the direction of further research. For example, Wang et al. [36] changed the YOLOv4 backbone from CSPDarknet53 to MobileNetv3, and used depth separation convolution instead of ordinary convolution in the feature fusion stage to reduce the amount of model parameters. The size of the model is greatly reduced without reducing the accuracy, and the detection speed is improved. With no significant reduction in accuracy, lighter and faster models will definitely be the future trend.

Individuals and organizations involved in rice pest monitoring and management will benefit from this study, e.g., rice farmers, agronomists, and pest control companies. The proposed YOLO-GBS model can assist in the automatic monitoring and counting of rice pests, which can help farmers and agronomists make more informed decisions on pest management strategies. Pest control companies can also utilize this technology to enhance their pest detection and control services, leading to more efficient and effective pest control practices. Overall, the study’s findings can benefit the agricultural industry by improving pest management practices and reducing economic losses caused by rice pest damage. In the future, the combination of our proposed method with additional aspects, such as gender classification [37], could provide even more accurate and effective decision-making data support to personnel involved in protecting crops. This would be critical in improving crop protection measures and minimizing damage caused by pests.

## 5. Conclusions

In this study, seven kinds of rice pests were taken as the research object, an improved model named YOLO-GBS was proposed based on YOLOv5s—where GC attention mechanism and an additional detection head were introduced to YOLOv5s—BiFPN was used to replace PANet, and Swin Transformer from deep network was used to replace convolution. The improved network was trained on the data set containing 7 kinds of rice pests and the complete IP102 data set with 102 classes. Conclusions can be drawn as follows:

1. Based on the self-made rice pest data set with seven categories, the mean average precision of the improved YOLO-GBS target detection algorithm is 79.8%, which is 5.4% higher than the original YOLOv5s. It can also achieve better detection results in complex scenes.
2. By comparing the improved YOLO-GBS with common target detection algorithms such as YOLOv3, Faster RCNN, SSD, etc., the results show that YOLO-GBS has excellent performance in detection accuracy and time. It has an incredibly good comprehensive performance, meeting the requirements of real-time detection accuracy and the speed of rice pests.
3. This study discusses the detection performance of YOLO-GBS on large-scale pest data sets. The experimental results show that the improved model has good robustness and generalization performance, with the possibility of further applications to other crop pest detection.

## Figures and Tables

**Table 1:** Results of ablation experiment.

Methods	Precision (%)	Recall (%)	mAP (%)
YOLOv5s (baseline)	71.2	68.4	74.4
YOLOv5s + P6	71.9	70.7	75.1
YOLOv5s + P6 + BiFPN	72.8	71.2	75.4
YOLOv5s + P6 + BiFPN + GC	73.2	72.7	76.5
YOLO-GBS (previous + Swin Transformer)	73.8	75.1	79.8

**Table 2:** Comparison of the model performance.

Methods	mAP (%)	Detection Time (ms)
SSD300	68.3	11.9
YOLOv3-tiny	69.6	1.9
YOLOv3	74.6	7.6
Faster RCNN	75.4	18.3
YOLO-GBS	79.8	3.2

**Table 3:** Comparison of detection effects of different networks in IP102 dataset.

Methods	mAP (%)
Faster RCNN [19]	47.9
FPN [19]	54.9
SSD300 [19]	47.2
RefineDet [19]	49.0
YOLOv3 [19]	50.6
YOLOv5s	51.4
YOLO-GBS	55.7

## Data Availability

Publicly available datasets were used in this study. This data can be found here: https://github.com/xpwu95/IP102. Additional supplements are available on request from the corresponding author.
